# Human narcolepsy is linked to degeneration of both locus coeruleus and hypocretin neurons

**DOI:** 10.1038/s41467-026-70899-x

**Published:** 2026-03-28

**Authors:** Thomas C. Thannickal, Ming-Fung Wu, Marcia E. Cornford, Jerome M. Siegel

**Affiliations:** 1 Neuropsychiatric Institute and Brain Research Institute, University of California, Los Angeles, Los Angeles, CA USA; 2 Neurobiology Research, Veterans Administration Greater Los Angeles Healthcare System, North Hills, CA USA; 3 Department of Pathology, Harbor-UCLA Medical Center, Torrance, CA USA

**Keywords:** Sleep disorders, Cellular neuroscience

## Abstract

Earlier studies led to the conclusion that human narcolepsy with cataplexy was correlated with the loss of hypocretin (Hcrt = orexin) neurons in the hypothalamus. We now report that individuals having narcolepsy with cataplexy also have an average 46% loss in the number and an 18% increase in the size of brainstem norepinephrine neurons in the locus coeruleus (LC), based on our analysis of 11 brains of individuals having narcolepsy with cataplexy. Microglial clustering around Hcrt neurons in the hypothalamus and around noradrenergic neurons in the locus coeruleus of individuals having narcolepsy with cataplexy indicates microglial involvement in the degeneration of both of these neuronal groups.

## Abstract (web summary)

Prior work indicated that loss of neurons in the hypothalamus containing hypocretin was responsible for human narcolepsy. Here, authors show that all narcolepsy patients also have a loss of noradrenergic neurons in the brainstem locus coeruleus.

## Introduction

A study in mice blocking the synthesis of hypocretin (Hcrt = orexin), a forebrain hypothalamic peptide, noted symptoms somewhat resembling those of narcolepsy^1^. This led to anatomical studies of the hypothalamus of human narcoleptics with cataplexy, revealing a ~90% loss of neurons containing Hcrt^2,3^, and to treatment of narcolepsy by administering Hcrt agonists^4^, supplementing the traditional treatment of narcolepsy with a variety of stimulants^5,6^.

Our report in 2000 of hypocretin neuron loss in narcolepsy was based on the brains of 4 people having narcolepsy with cataplexy (NT1)^2,3^. Peyron et al.’s^7^ report in 2000 on hypocretin neuron loss in narcolepsy was based on the brains of 2 people with narcolepsy. Our current report includes analyses of the brains of 11 patients having narcolepsy with cataplexy (NT1). All 11 subjects showed a loss of both Hcrt neurons in the hypothalamic forebrain and of noradrenergic neurons in the brainstem locus coeruleus.

In contrast to the original anatomical findings on small numbers of subjects, the level of hypocretin in cerebrospinal fluid (CSF) has now been assayed in hundreds of patients diagnosed with narcolepsy by behavioral criteria, including the “gold standard” multiple sleep latency test^8^. A surprising finding is that 15–30% of people diagnosed as having “narcolepsy with cataplexy (NT1)”, have absolutely normal CSF hypocretin levels^9–30^. This finding is not consistent with the popular hypothesis that the loss of hypocretin causes both sleepiness and cataplexy, the principal symptoms of narcolepsy. The CSF hypocretin levels in people diagnosed with “narcolepsy without cataplexy (NT2),” who constitute the majority of people diagnosed with narcolepsy, do not have hypocretin levels differing from normal controls^9–30^. Again, this is not consistent with the hypothesis that hypocretin loss causes narcolepsy.

Two speculations attempting to explain this paradox include the idea that perhaps some hypocretin neurons, while intact, are not releasing the hypocretin peptide. Another is that a hypocretin receptor abnormality exists in people with narcolepsy. But these speculations have not been supported by any convincing evidence.

A third speculation has been that damage to some other unspecified transmitter system may be causing narcolepsy. This is the topic of the present report. We have found that all of the brains we have acquired from narcolepsy with cataplexy patients (NT1) have a major loss of locus coeruleus neurons, whereas no control brains have this loss. It is well established that locus coeruleus neurons project both rostrally as part of the ascending arousal system and caudally to regulate muscle tone^31–37^, suggesting that their dysfunction could cause both of the major symptoms of narcolepsy.

## Results

### Brainstem pathology in the locus coeruleus of narcolepsy with cataplexy patients

**Fig. 1:**
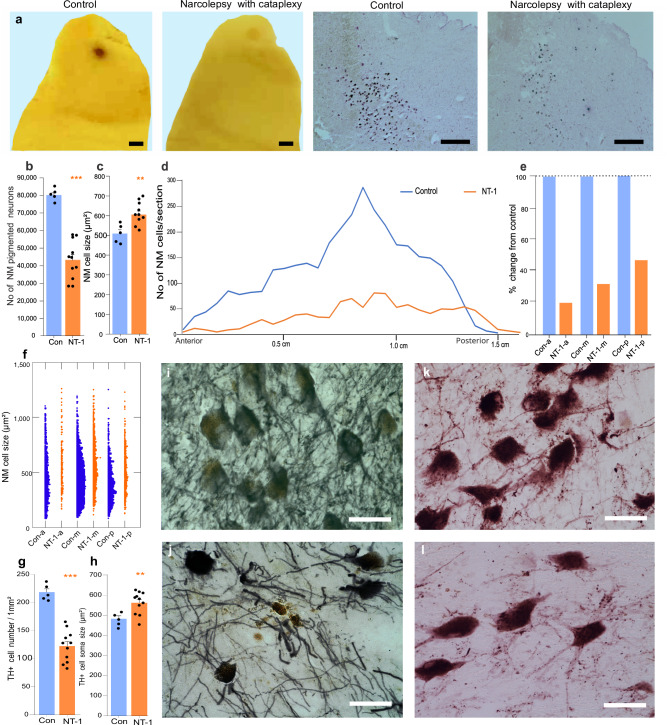
Neuromelanin and norepinephrine cell loss in the locus coeruleus of narcolepsy with cataplexy patients. **a** Far left, unstained brainstem block of control patient shows the dark spot of the locus coeruleus (LC) neurons, 2–2.5 mm in diameter. Left, center, similar unstained block shows faded LC spot in NT1 patients. Right, center, cresyl violet-stained microscopic image from control shows neuromelanin (NM) containing LC neurons. Far right, NT1 patients show reduced number of NM neurons in the LC compared to control subjects. Fourth ventricle is to the right in each of the top 4 images. **b** Stereological calculations show a significant reduction in the number of NM pigmented cells in NT1 (*n* = 11) compared to controls (*n* = 5) (*t*-test, df = 14, *t* = 7.32, *P* = 0.001). **c** Neuromelanin cell size was significantly larger in NT1 patients compared to controls (*t*-test, df = 14, *t* = 3.24, *P* = 0.01). **d** Neuromelanin cell distribution from anterior to posterior part of LC of control and NT1 patients. **e** The percentage changes in number within each subregion in narcoleptic compared to control: (a) anterior, (m) middle, and (p) posterior in control (blue) and narcolepsy with cataplexy (orange) subjects. **f** NM cell size distribution from anterior, middle and posterior parts of the LC in control and NT1 subjects. **g** In NT1 patients, TH+ cell density is significantly reduced compared to control (*n* = 11 and 5 respectively, *t*-test, *t* = 7.32, df = 14, *P* = 0.001). **h** Size of the TH+ neurons are larger in NT1 than in controls (*t*-test, *t* = 2.91, df = 14, *P* = 0.01). **i**, **j** Images of NM pigmented neurons in control (Top) and NT1 (bottom) with H&E counterstaining. **k**, **l** Immunohistochemistry images of DBH+ cells from control (top) NT1 (bottom). ^**^P < 0.01, ^***^P < 0.001. Data expressed as mean ± SEM. Scale bar 2 mm and 500 µm in Fig. **a**. Scale bar 50 µm in Fig. **i–l**. Con – control, Con-a - anterior, Con–m - middle, Con–p - posterior, DBH – dopamine beta-hydroxylase, NM - neuromelanin pigmented neurons, NT1 - narcolepsy with cataplexy, NT1-a - anterior, NT1-m - middle, NT1-p - posterior, TH+- tyrosine hydroxylase.

We conducted a histological analysis of the locus coeruleus (LC) of humans with narcolepsy, analyzing the LC of 11 narcolepsy with cataplexy patients (NT1) and 5 control subjects (Table 1). We used hematoxylin-eosin (H&E) staining to assess the number and size of neuromelanin (NM) pigmented^38^ norepinephrine containing neurons (Fig. 1a–f). Neuromelanin is a dark brown pigment that forms in norepinephrine and dopamine neurons and increases with age in humans, reaching adult levels in the teenage years^38^. We found that all 11 brains of humans having narcolepsy with cataplexy (NT1) had a loss of norepinephrine neurons in LC, in addition to the previously reported loss of forebrain Hcrt neurons^2,3,7^.

**Table 1:** The characteristics of human subjects and neuromelanin pigmented cells number and size

Human narcolepsy with cataplexy												
NT1	Age		Sex	Cataplexy onset		Disease duration (years)	Cause of death	NM cell number	% cell loss	NM cell size (μm^2^)	Medications	Neuropathology report
1	79		M	18		61	Cardiomyopathy, cancer	32,449	59.24	541.81	modafinil	Normal neocortical and hippocampal cellularity.
2	72		M	20		52	CH, lung cancer	38,304	51.88	587.41	methamphetamine	Early mild Alzheimer's pathology in hippocampus and frontal cortex.
3	90		M	18		72	Congestive heart failure	28,128	64.66	599.00	methamphetamine	No indication of Alzheimer’s disease.
4	79		M	21		58	Coronary heart disease Cancer- prostate and lung	38,800	51.26	525.35	methamphetamine	Alzheimer's pathology in frontal, temporal and parietal cortex. Neuritic plaques and amyloid arteriolar vascular deposition with moderate numbers of intraneuronal fibrillary inclusions.
5	90		F	18		72	Cancer – esophagus congestive heart failure kidney cancer	56,820	28.62	546.69	protriptyline	Dendritic bodies in cerebellar grey matter, cerebral cortex and medulla. No Lewy bodies found.
6	94		F	21		73	Cancer, COPD	56,088	29.54	699.16	dextroamphetamine	Amyloid plaques in hippocampus and temporal neocortex.
7	61		F	19		42	Pneumonia	44,654	43.90	683.45	methamphetamine	NA
8	68		F	23		45	COPD, pneumonia	56,720	28.75	662.45	NA	NA
9	88		F	13		70	Cardiomyopathy	27,981	64.85	583.47	dextroamphetamine	Minimal Alzheimer's pathology, plaques and NFTs in frontal cortex.
10	91		F	16		75	Heart disease	47,136	40.79	602.34	modafinil	Few NFTs in lateral frontal cortex with no plaques.
11	76		F	30		46	NA	43,776	45.01	627.33	NA	Minimal Alzheimer's pathology.
Control subjects												
1	71	M					Myocardial infarction	84,288		454.86		
2	82	M					Myocardial infarction	80,622		517.20		
3	80	M					Sepsis, pneumonia	74,760		564.37		
4	83	F					Pneumonia	78,695		542.10		
5	72	F					Acute myocardial infarction	79,688		467.07		

*CH* clonal hematopoiesis, *COPD* chronic obstructive pulmonary disease, *NA* not available, *NM* neuromelanin pigmented cells, *NFT* neurofibrillary tangles, *NT1* narcolepsy with cataplexy.

The substantial reduction in the number of neuromelanin pigmented norepinephrine LC neurons in narcolepsy with cataplexy patients (NT1) can be seen in unstained tissue in Fig. 1. Figure 1a, far left vs. middle left, shows faded “locus coeruleus spot” in a patient having narcolepsy with cataplexy, with 46% fewer neuromelanin pigmented norepinephrine neurons compared to control subjects (Fig. 1a, hematoxylin-eosin stained middle right vs far right). Figure 1b shows a loss range of 28−68% (*t* = 8.52, df = 14 and *P* = 0.001). Furthermore, the neuromelanin pigmented norepinephrine neurons in narcolepsy with cataplexy patients (NT1) were 18% larger than those in controls (Fig. 1c, *t* = 7.32, df = 14, and *P* = 0.01). Figure 1d–f shows the distribution of neuromelanin pigmented neurons in the anterior-to-posterior dimension of the LC of a narcolepsy with cataplexy patient (90 yrs, M) and a control subject (82 yrs, M). We found that the greatest loss of norepinephrine cells (averaging approximately 80%) occurred in the anterior portion of the LC (Fig. 1e). There was no significant correlation between the percentage loss of neuromelanin-pigmented norepinephrine neurons and age of cataplexy onset (*r* = 0.28, *n* = 11, *P* = 0.39). There was a significant sex difference in the number (female: 47596.4 ± 3924.9, male: 34420.3 ± 2545.3; *t* = 2.34, df = 9 and *P* = 0.04) but not in the size (*t* = 2.11, df = 9 and *P* = 0.06) of neuromelanin pigmented neurons in the narcolepsy with cataplexy patients.

We then performed an immunohistochemical analysis using a tyrosine hydroxylase (TH) antibody to visualize the rate-limiting enzyme in catecholamine synthesis (Fig. 1g–j) (Supplementary Table 1). 95% of the TH-containing LC neurons also contain neuromelanin^39^. The loss of TH-positive neurons in narcolepsy with cataplexy patients was 44% (*t* = 8.12, df = 14, *P* = 0.001), and there was a significant increase in the size of TH-positive cells in narcolepsy with cataplexy patients compared to controls (*t* = 7.21, df = 14, *P* = 0.01). The loss of TH+ neurons was not significantly correlated with the age of onset of cataplexy (*r* = 0.35, *P* = 0.28). The number and size of TH neurons in narcolepsy groups did not differ between males and females (*t* = 0.28, df = 9, *P* = 0.78: *t* = 0.15, df = 9, *P* = 0.88). Dopamine β-hydroxylase (DBH), the enzyme that converts dopamine into norepinephrine, was used to identify noradrenergic locus coeruleus neurons. Figure 1k, l shows the DBH-stained cells in control and in narcolepsy with cataplexy patients.

Surviving LC norepinephrine cells in narcoleptics exhibited minimal neuronal inclusions, unlike the LC neurons in Alzheimer’s and Parkinson’s disease. The absence of neuronal inclusions in surviving LC neurons suggests that cell loss in narcolepsy results from a selective, likely immune-mediated, process^40,41^ rather than the degenerative pathology characteristic of Alzheimer’s or Parkinson’s disease.

### Microglial and neurodegenerative markers in the LC of narcolepsy with cataplexy patients

**Fig. 2:**
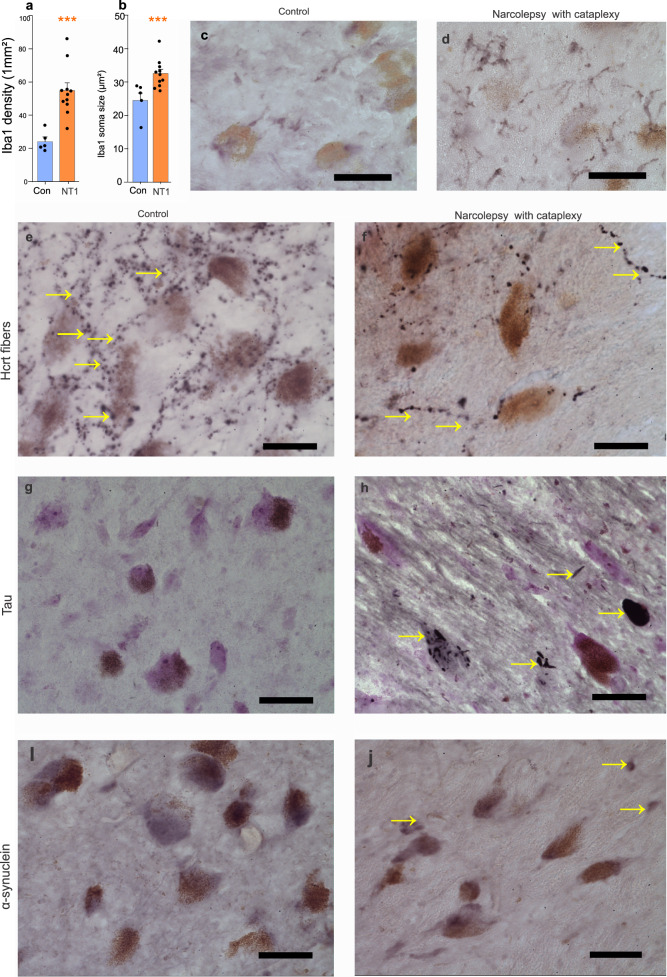
Distribution of microglia, Hcrt fibers, alpha-synuclein, and Tau proteins in the LC of narcolepsy with cataplexy patients. **a** Immunohistochemistry of Iba1(ionized calcium-binding adaptor molecule) shows a significant increase in the number of microglia in the locus coeruleus of narcolepsy with cataplexy patients (NT1) compared to control (Con) subjects (*t*-test, *n* = 5 in control and 11 in narcolepsy with cataplexy groups, df = 14, *t* = 4.29, *P* = 0.001). **b** Iba1 cell size also significantly increased (*t*-test, *t* = 3.41, df = 14, *P* = 0.001), (5 control and 11 NT1 subjects). ^***^P < 0.001. Data expressed as mean ± SEM. **c** Representative immunohistochemical images of Iba1 in control and **d** narcolepsy with cataplexy subject. **e**, **f** Distribution of Hcrt fibers in the LC of control and narcolepsy with cataplexy subject (*n* = 5 control and *n* = 11 NT1 patients). As might be expected, Hcrt axons, originating in Hcrt neurons in the hypothalamus are greatly reduced in humans with narcolepsy because of the death of hypothalamic Hcrt neurons. **g**, **h** Representative immunological staining of Tau protein (black) deposits in the LC of control and narcolepsy with cataplexy patients (*n* = 5 control and *n* = 11 NT1 patients). **i**, **j** Representative mages of α-synuclein distribution in the LC of control and narcoleptic subjects (*n* = 5 control and *n* = 11 NT1 patients). α-synuclein and Tau are increased in narcoleptics. Scale bar 50 µm.

We found a 118% increase in microglial density in the LC of narcolepsy with cataplexy patients compared to controls (Fig. 2a, *t* = 4.45, df = 14, *P* = 0.001). Furthermore, there was a notable enlargement of LC microglia, which were 33% larger than LC microglia in human control brains (Fig. 2b, *t* = 3.41, df = 14, *P* = 0.001) (Supplementary Table 1). The distribution of Iba1-positive cells in both control and narcolepsy with cataplexy (NT1) subjects is shown in Fig. 2c, d. There was no significant correlation between the increased numbers of Iba1 and the age of disease onset (*r* = 0.39, *n* = 11, *P* = 0.24) or of disease duration (*r* = 0.41, *n* = 11, *P* = 0.22). No statistically significant sex-based differences were observed in the Iba1 changes in number or size in narcolepsy with cataplexy patients (NT1) (*t* = 0.41, df = 9, *P* = 0.69; *t* = 1.96, df = 9, *P* = 0.08). The increase in the number of microglial cells in the LC of human narcoleptics suggests a neuroinflammatory process^40,41^. Although the narcolepsy patients had differing drug treatments (Table 1), all human narcolepsy patients showed a similar loss of LC neurons. The neuropathology report for narcolepsy with cataplexy patients (NT1) indicated that 40% of them exhibited some degree of Alzheimer’s pathology (Table 1). The number of Hcrt axons was lower in the LC of narcolepsy with cataplexy (NT1) patients compared to controls (Fig. 2e, f), consistent with the reduction in the number of Hcrt neurons in narcolepsy. We investigated the presence of α-synuclein and tau protein deposits in the LC of narcolepsy with cataplexy (NT1) and control subjects. Although little or no tau or α-synuclein deposits were found in control subjects, tau (Fig. 2g, h) and α-synuclein (Fig. 2i, j) deposits were observed in the LC of narcolepsy with cataplexy (NT1) patients.

### Neuronophagy in the hypothalamus and LC of narcolepsy with cataplexy patients

**Fig. 3:**
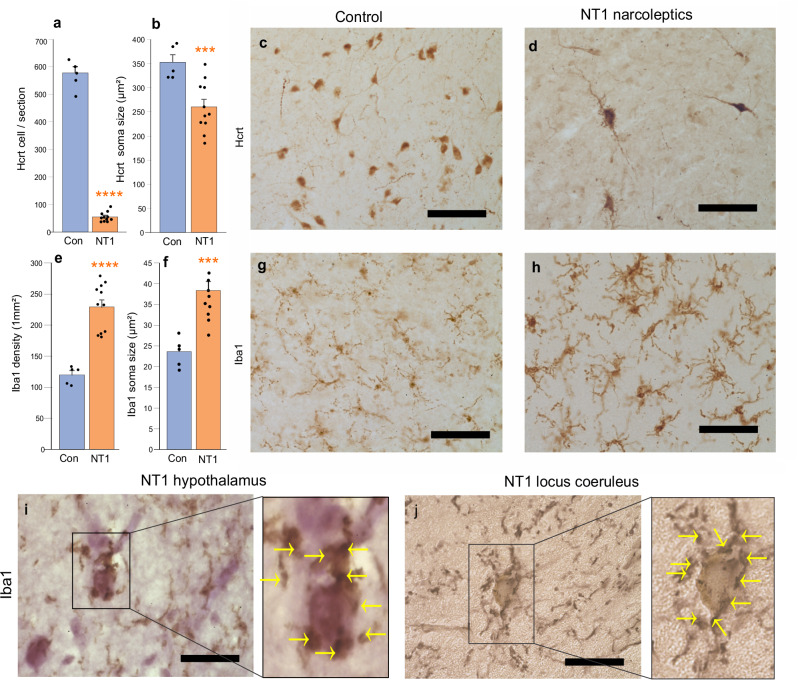
Neuronophagy in the hypothalamus and LC of narcolepsy with cataplexy patients. **a** In narcolepsy with cataplexy patients (NT1, *n* = 11), 90% of Hcrt cells were lost compared to control (*n* = 5), *t*-test, *t* = 31.15, df = 14, *P* = 0.0001). **b** The size of the surviving Hcrt neuros were significantly reduced (NT1 *n* = 11, control *n* = 5; *t*-test, *t* = 3.65, df = 14, *P* = 0.001) (**a**–**d**). **c**, **d** The Hcrt distribution in control and narcolepsy with cataplexy patients. **e** The number of microglia significantly increased in the hypothalamus of narcolepsy with cataplexy patients (*n* = 5 control and *n* = 11 NT1 patients; *t*-test, *t* = 6.18, df = 14, *P* = 0.0001). **f** Significant increase in the size of the microglia in narcolepsy with cataplexy subjects (*n* = 5 control and *n* = 11 NT1; *t*-test, *t* = 4.22, df = 14, *P* = 0.001). **g**, **h** The microglial distribution in control and narcolepsy with cataplexy patients. **i** Microglial clustering around neuronal soma in the hypothalamus of narcolepsy with cataplexy. **j** A neuromelanin pigmented neuron is surrounded with microglia in the LC of narcolepsy with cataplexy. Data expressed as mean ± SEM. ^***^P < 0.001, ^****^P < 0.0001. Scale bar 50 µm.

The number of Hcrt neurons in the hypothalamus of narcolepsy with cataplexy (NT1) patients was reduced by 90% compared to controls (Fig. 3a, c, d; *t* = 31.15, df = 14, *P* = 0.0001). Hcrt neurons were 26.5% smaller in narcolepsy with cataplexy (NT1) patients compared to controls (*t* = 3.65, df = 14, *P* = 0.001) (Fig. 3b–d) (Supplementary Table 2). In narcolepsy with cataplexy (NT1) subjects, no difference was observed in the number (*t* = 0.04, df = 9, *P* = 0.96) and size (*t* = 0.45, df = 9, *t* = 0.66) of Hcrt neurons in male vs. female patients. Hypothalamic Iba1 (ionized calcium-binding adaptor molecule), a marker for activated microglia, was significantly elevated (Fig. 3e–h), with a 90.8% increase in cell number (*t* = 6.18, df  = 14, *P* = 0.0001) and a 62.3% enlargement in soma size (*t* = 4.22, df = 14, *P* = 0.001) in narcolepsy with cataplexy (NT1) patients (Supplementary Table 2). There was no significant difference between males and females in either the number (*t* = 1.02, df = 9, *P* = 0.33) or the size (*t* = 0.13, df = 9, *P* = 0.89) of microglial cells in narcolepsy with cataplexy subjects. Microglial clustering was observed in the hypothalamus (Fig. 3i) and in the LC of narcolepsy with cataplexy (NT1) patients (Fig. 3j). Norepinephrine cell loss in the LC did not correlate significantly with Hcrt neuron loss in the hypothalamus (*r* = 0.19). Likewise, the magnitude of microglial alterations in the LC showed no significant association with microglial changes in the hypothalamus (*r* = 0.25).

### The number of norepinephrine neurons is not affected by the loss of Hcrt neurons in narcoleptic mice, or by the receptor mutation in canine genetic narcolepsy

**Fig. 4:**
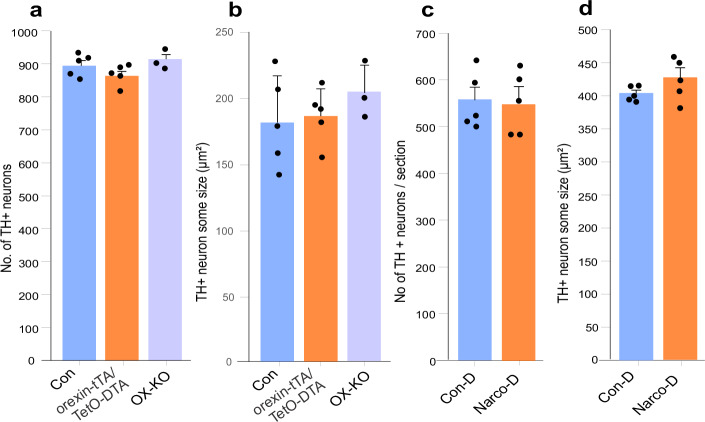
Norepinephrine neurons in mice and dog models of narcolepsy. **a** The number of TH+ cells in the LC of control, OX-KO and orexin-tTA/TetO-DTA mice. We saw no significant changes in the number of TH+ neurons in the LC in any of these models compared to controls (*n* = 5), OX-KO mice (*n* = 3; *t*-test, *t* = 0.85, df = 6, *P* = 0.42) and DTA group (*n* = 5; *t*-test, *t* = 0.17, df = 8, *P* = 0.16). **b** There is no difference in TH+ cell size between control and OX-KO mice (*n* = 3 OX-KO, *t*-test, *t* = 0.99, df = 6, *P* = 0.36) or control and DTA mice (*n* = 5 DTA group; *t*-test, *t* = 0.27, df = 8, *P* = 0.79). **c** Narcoleptic Doberman pinschers show no difference from controls in the number of TH+ neurons in the LC (*n* = 5 in each group; *t*-test, *t* = 0.22, df = 8, *P* = 0.83). **d** TH+ cell size also did not differ between narcoleptic and control dogs (*n* = 5 control and *n* = 5 narcoleptic dogs; *t*-test, *t* = 1.45, df = 8, *P* = 0.18). Data expressed as mean ± SEM.

We used two distinct mouse models of narcolepsy. One model was orexin-tTA/TetO-DTA (OX-DTA) in which Hcrt neuron degeneration can be initiated by removal of doxycycline (DOX) from the diet^42^. Removing DOX food for 1 month (and replacing it with conventional mouse food) caused an 87% loss of Hcrt neurons in these animals (Supplementary Fig. 1). The second mouse model of narcolepsy is mice in which the Hcrt peptide cannot be synthesized (orexin knockout - OX-KO). We evaluated the changes in LC norepinephrine cells in these two narcoleptic models, quantifying immuno-stained TH+ cells (Fig. 4a, b). We saw no significant changes in the number (OX-DTA: *t* = 1.52, df = 8, *P* = 0.17, OX-KO: *t* = 0.85, df = 6, *P* = 0.42) or size (OX-DTA: *t* = 0.27, df = 8, P = 0.79, OX-KO: *t* = 0.99, df = 6, *P* = 0.36) of the TH+ cells in the LC in these two animal models of narcolepsy compared to wild-type control mice. In addition to the two mouse models, we also examined changes in norepinephrine cells in the LC of narcoleptic dogs, a genetic narcoleptic disorder that occurs without any prenatal or postnatal manipulation^43,44^ (Supplementary Table 3). No changes were observed in the norepinephrine cell number (*t* = 0.22, df = 8, *P* = 0.83) and size (*t* = 1.45, df = 8, *P* = 0.18) compared to breed matched, non-narcoleptic, control dogs (Fig. 4c, d). Thus, the loss of norepinephrine in locus coeruleus that we describe here as a feature of human narcolepsy with cataplexy (NT1) is not caused by the Hcrt loss or by Hcrt receptor changes^45^ in these animal models.

### Sodium oxybate effect on LC norepinephrine and microglia cells

**Fig. 5:**
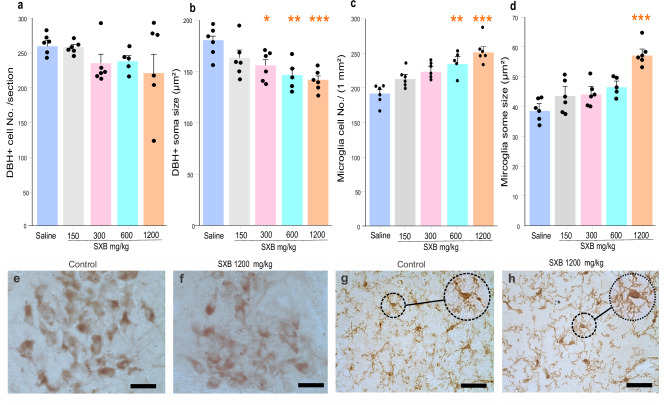
Effect of sodium oxybate (SXB) on mice LC norepinephrine (DBH+) cells and microglia. **a**, In comparison to the saline group (*n* = 6), the number of DBH+ cells was not significantly affected by various doses of SXB (150 mg/kg, *n* = 6, 300 mg/kg, *n* = 6, 600 mg/kg, *n* = 5; 1200 mg/kg, *n* = 6) (ANOVA, df = 4, 24, *F* = 1.27, *P* = 0.31, two-sided). **b** The cell size of DBH+ decreased with higher concentrations of SXB (ANOVA, df = 4, 24, *F* = 6.65, *P* = 0.001, two-sided). DBH+ cell size was significantly different from that of the saline controls at 300 mg/kg (13.7%, *P* = 0.047), 600 mg/kg (18.9%, *P* = 0.005) and 1200 mg/kg SXB (21.6%, *P* = 0.0001), all Tukey post-hoc. **c** Microglia cell number increased with higher doses of SXB (ANOVA, df = 4, 24, *F* = 7.81, *P* = 0.001, two-sided). The increase in the number of microglia cells is significant (Tukey post-hoc) at the dose of 600 mg/kg (22.08%, *P* = 0.012) and 1200 mg/kg SXB (31.7%, *P* = 0.001). **d** The size of microglia increased with increasing doses of SXB (ANOVA, df = 4, 24, *F* = 7.37, *P* = 0.001, two-sided). A significant increase was found at 1200 mg/kg (*P* = 0.001, Tukey post-hoc). **e** Histological images of DBH + neurons with saline and **f** 1200 mg/kg SXB. **g** Images of microglia with saline and **h** 1200 mg/kg SXB treated mice. Data expressed as mean ± SEM. ^*^P < 0.05, ^**^P < 0.01, ^***^P < 0.001, compared to saline group. Con-control, Con-D -control dog, LC- locus coeruleus, Narco-D- narcoleptic dog, OX-KO-orexin knockout mice, TH- tyrosine hydroxylase. DBH – dopamine beta- hydroxylase, SXB - sodium oxybate, Scale bar 50 µm.

Sodium oxybate (SXB) is an effective treatment for human narcolepsy with cataplexy^46,47^. We conducted a dose-response study of the effect of SXB on the LC in wild-type mice^48^. We stained norepinephrine neurons with dopamine beta hydroxylase (DBH) and the Iba1 stain to quantify the number and size of microglial cells. The doses tested were 150 mg/kg, 300 mg/kg, 600 mg/kg, and 1200 mg/kg (Supplementary Table 3). SXB treatment had no significant effect on the number of DBH+ neurons (Fig. 5a, ANOVA, df = 4, 24, *F* = 1.27, *P* = 0.31), but there was a dose-dependent decrease in the size of DBH neurons with SXB (Fig. 5b, ANOVA, df = 4, 24, *F* = 6.65, *P* = 0.001). Tukey post-hoc comparisons indicated a significant size reduction as compared to saline controls with 300 mg/kg (13.7%, *P* = 0.047), 600 mg/kg (18·9%, *P* = 0.005) and 1200 mg/kg SXB (21.6%, *P* = 0.001). Figure 5e, f shows DBH+ neurons from saline and 1200 mg/kg SXB-treated mice.

SXB significantly increased the number of microglia cells in locus coeruleus (ANOVA, df = 4, 24, *F* = 7.81, *P* = 0.001) (Fig. 5c). The increase in the number of microglia cells was significant (Tukey post-hoc) at the doses of 600 mg/kg (22·08%, *P* = 0.012) and 1200 mg/kg SXB (31.7%, *P* = 0.001). The size of the microglia was also significantly increased with higher doses of SXB (ANOVA, df = 4.24, *F* = 7.37, *P* = 0.001, Fig. 5d). A significant increase was found at 1200 mg/kg SXB (48.28%, *P* = 0.001). Figures 5g, h shows microglial cells from saline and 1200 mg/kg SXB-treated mice. The precise mechanism by which these changes affect symptoms is unknown.

## Discussion

The activity of Hcrt neurons is not consistent with the symptoms that might be predicted from their loss in narcolepsy, i.e., their activity is not correlated with increased alertness or muscle tone, but rather is related to pleasure^49–51^. Hcrt neurons are maximally active when rats are moving forward, grooming or eating. However, when a rat recoils from the first piece of chicken it has seen, Hcrt neurons are inactive, despite strong EEG alerting and strong motor activity (See Supplementary Movie 1).

Hypocretin release is greatly elevated when normal Doberman dogs are playing in a yard, but it is not elevated by comparable levels of motor activity on a treadmill, suggesting that it was play, rather than motor activity, that was responsible for elevated levels of Hcrt^50^. We had the opportunity to measure Hcrt release in the human brain and saw a similar pattern; Hcrt release was maximal when subjects were engaged in pleasant social interactions. But Hcrt release was at its very lowest level during pain, despite elevated muscle tone and EEG alerting^51^. This is not consistent with the hypothesis that Hcrt levels are simply correlated with EEG activation.

We recently found that opioid dependence causes increased Hcrt production in Hcrt neurons and shrinkage of these Hcrt neurons in both human heroin addicts and mice^52^. Repeated morphine exposure impairs spike generation in a subpopulation of Hcrt neurons^53^. We further found that blocking Hcrt receptors with the dual Hcrt receptor blocker suvorexant prevents opioid induced neuroanatomical, microglial and behavioral changes without diminishing opioid analgesia^54^. These changes are consistent with Hcrt neurons involvement in opioid addiction, but not in opioid analgesia^54^.

In contrast to Hcrt neurons, locus coeruleus neurons are known to be active during alerting and periods of high levels of muscle tone. Locus coeruleus neurons have both ascending axons contributing to EEG arousal, and descending axons elevating muscle tone^31,36,55–57^. We find that a cessation of firing in locus coeruleus neurons is tightly linked to the loss of muscle tone in REM sleep and in cataplexy^37,58^ (see Supplementary Fig. 2). Therefore, the loss of locus coeruleus neurons in narcolepsy can explain both the EEG and motor symptoms of narcolepsy, i.e., excessive sleepiness and cataplexy. In contrast, the loss of Hcrt neurons can explain the absence of opioid dependence and the increased incidence of depression in narcolepsy^59–65^.

We see pathological changes in the LC of all the brains of humans with narcolepsy that we have examined, but none in the narcoleptic OX-DTA^42^ or OX-KO mice^1^, or in the narcoleptic dogs^43,66^, despite the descending projection of hypothalamic Hcrt neurons to the region of LC^67^. Thus, loss of hypothalamic Hcrt neurons does not cause the loss of locus coeruleus neurons that we have seen in human narcolepsy with cataplexy. The findings that identified the loss of Hcrt neurons as a feature of human narcolepsy with cataplexy^3,7^, should not obscure the differences between canine, murine and human narcolepsy. Although both murine and human narcolepsy are correlated with the loss of Hcrt neurons, in this study, we find that human but not murine cataplexy is accompanied by the loss of neuromelanin-containing tyrosine hydroxylase neurons in locus coeruleus. Neuromelanin, which is present in the locus coeruleus neurons of adult humans, is not present in canine or rodent brains at any age^38^.

SXB’s therapeutic effect on narcoleptic symptoms in humans may be mediated by activation of the remaining norepinephrine neurons. SXB reduced TH immuno-intensity in LC^48^ in mice. SXB has been shown to improve night-time sleep quality and reduce symptoms of both excessive daytime sleepiness and cataplexy in patients with narcolepsy. SXB is believed to increase the activity of the neurons with gamma-aminobutyric acid type B (GABA-B) receptors, in regions that regulate sleep-wake homeostasis^46–48^. In animals, high doses of SXB have been shown to induce a hypersynchronous EEG state^68,69^.

Reboxetine, a selective noradrenergic reuptake inhibitor, has been found to be highly effective in treating human narcolepsy^70^. SXB is thought to act in part by boosting the activity of the noradrenergic neurons of the locus coeruleus^71^. A recent comprehensive study evaluating the relative effectiveness of all current pharmacological treatments for narcolepsy showed that solriamfetol (Sunosi), a dopamine and norepinephrine reuptake inhibitor, was more effective than any other current treatment for narcolepsy, including modafinil, sodium oxybate and pitolisant. Solriamfetol reduced Epworth sleepiness scale scores and prolonged sleep latency more than any other treatment for narcolepsy^72^. These data are consistent with our finding herein of a major loss of noradrenergic neurons in human narcolepsy.

Tau and α-synuclein deposits in the LC of narcolepsy with cataplexy patients suggest that narcolepsy with cataplexy shares some neurodegenerative pathology with Alzheimer’s and Parkinson’s diseases, despite an earlier disputed claim that narcolepsy decreased the incidence of Alzheimer’s^73^.

Pathological alterations in the hypothalamus of narcolepsy with cataplexy patients are not limited to the hypocretin system. Additional changes include a reduction of corticotropin-releasing hormone (CRH)-producing neurons in the paraventricular nucleus of the hypothalamus^74^. In contrast to this neuronal loss, an increase in histaminergic neurons has been reported in the forebrain of narcolepsy with cataplexy patients^75,76^. It is likely that neuroscientists are just beginning to fully describe the differences between the brains of people with narcolepsy and control populations. One needs to attempt to differentiate between the changes causing narcolepsy, the changes resulting from degenerative changes causing narcolepsy, and the changes caused by long-term drug treatment.

In this respect the evolution of our understanding of the neuropathology of narcolepsy appears to have a parallel to early work on Alzheimer’s, which was first reported to be due to a selective loss of cholinergic neurons^77^, but which is now known to be characterized by the loss of glutamatergic, adrenergic, serotonergic, dopaminergic and locus coeruleus neurons^31^, and by widespread amyloid plaques, tau protein tangles and neuroinflammation. Indeed, brain size is reduced by about 20% in Alzheimer’s disease^78^.

Functional magnetic resonance imaging (fMRI) of the brainstem revealed reduced neuromelanin levels in the locus coeruleus of human narcolepsy patients^79^, suggesting that this non-invasive technique could be extremely helpful in the diagnosis and treatment of human narcolepsy. Neuromelanin levels in LC reach their maximum in the teenage years, the time when most narcolepsy symptoms begin^79,80^.

Differences between the symptoms in the three animal models of narcolepsy (OX- KO, OX-DTA, canine narcolepsy) and human narcolepsy would be expected. Further quantitative research, with more precise measures of motor function, and particularly muscle tone control, may clarify the differences in the behavioral consequences of the neuroanatomical changes between these differing forms of narcolepsy.

Our findings of brainstem neurodegeneration in narcolepsy with cataplexy patients demonstrate that the “Hcrt loss” explanation of human narcolepsy is incomplete. Clustering of microglia in the hypothalamus and locus coeruleus indicates microglial involvement in the loss of hypocretin and norepinephrine neurons. Microglia maintain close interactions with neurons to regulate their function, and disruption of this network can contribute to neurodegeneration^81^. Investigating the role of microglia in narcolepsy offers an avenue to clarify the mechanisms underlying the loss of hypocretin and norepinephrine neurons reported here.

Our findings support the idea that the autoimmune mechanisms driving human narcolepsy extend to both forebrain hypocretin neurons and brainstem locus coeruleus norepinephrine cells. Considering the effects of damage to both regions may be essential for advancing our understanding of narcolepsy and for developing more effective therapeutic strategies targeted to the pathology of each patient.

## Methods

### Ethics declaration

Human postmortem brain tissue was obtained with permission and used in accordance with the relevant ethical boards at VA Greater Los Angeles Healthcare System. All animal procedures were approved by the Institutional Animal Care and Use Committees at the University of California, Los Angeles, and the Veterans Administration Greater Los Angeles Healthcare System.

### Human brainstem and hypothalamus immunohistochemical analysis

In this study, we examined post-mortem brain samples from eleven individuals with narcolepsy and five neurologically healthy controls. A summary of the brain sample characteristics is provided in Table 1. Narcolepsy diagnoses were based on the criteria set by the “American Academy of Sleep Medicine: International Classification of Sleep Disorders”. The control group consisted of individuals with no history of neurological disorders. Brainstem and hypothalamic tissues from narcolepsy and control subjects were obtained, with informed consent of donors and their family members, from the Department of Veterans Affairs Biorepository Brain Bank in Los Angeles and the Eunice Kennedy Shriver National Institute of Child Health and Human Development Brain Bank in Maryland.

Formalin-fixed brainstem tissues from control and narcoleptic human patients were equilibrated in 20% and 30% sucrose solutions. Following this, 40 µm coronal sections were prepared using a freezing microtome, and sections collected at one-in-twelve intervals. One series of sections was stained with Hematoxylin and Eosin (PK501, FD Neurotechnology Inc., Baltimore, MD) to highlight neuromelanin-pigmented cells in the locus coeruleus. Stereological techniques were employed to quantify the cell number, distribution, and size of these cells. Analyses were conducted using a Nikon E600 microscope with a three-axis motorized stage, video camera, Neurolucida interface, and Stereo Investigator software (MBF Bioscience, VT). The total number of neuromelanin-pigmented cells was then calculated.

The neuropathological changes in the LC were evaluated by using primary antibodies for Hcrt, TH, DBH, Iba1, Tau, and α-synuclein. Matched sections from the anterior, middle, and posterior parts of the LC were prepared based on adjacent H&E-stained sections of the human brainstem^31,82^. Sections from the hypothalamus were used for immunohistochemical studies of Hcrt and Iba1. Free-floating sections underwent antigen retrieval before immunostaining following the method outlined by Thannickal et al.^2,3^. The sections were incubated in 0.5% sodium borohydride for 30 min. After washes with phosphate-buffered saline (0.1 M, pH 7.4), sections were transferred to 0.5% hydrogen peroxide for 30 min, washed, and then heated at 80 °C for 30 min in a sodium citrate solution (10 mM, pH 8.5). The sections cooled to room temperature in the sodium citrate solution and were then washed with PBS. Following the PBS washes, the following primary antibodies were applied: Orexin A (1:10,000, rabbit polyclonal, Cat. No. H-003-30, Lot No. 01896-1, Phoenix Pharmaceutical Inc., CA), tyrosine hydroxylase (1:10,000, rabbit polyclonal, Cat. No. AB152, Lot No. 4224636, Millipore Sigma, MO), dopamine beta-hydroxylase (DBH; 1:5000, rabbit monoclonal, Cat. No. ab209487, Lot No. 1007675-38, Abcam), and α-synuclein (1:5000, rabbit monoclonal, Cat. No. ab51253, Lot No. GR3317474-4, Abcam). The sections were incubated in 1% normal goat serum in 0.01 M PBS for 2 h, followed by a 72-h incubation at 4 °C with the primary antibodies. For the secondary antibody, sections were incubated with biotinylated goat anti-rabbit IgG (1:500, PK-6101, Lot No. ZL0828 Vector Laboratories) for 2 h at room temperature, followed by avidin-biotin-peroxidase (1:300, PK-6101, Vector Laboratories) for 2 h. The tissue- bound peroxidase was visualized using a diaminobenzidine and nickel reaction (SK-4100, Vector Laboratories). For the DBH staining, Vector SK-4600 was used to achieve a purple color. Goat polyclonal Iba1 antibody (1:10,000, Cat. No ab5076, Lot No. GR3403958-1, Abcam) was used for microglia. Sections were incubated with secondary antibody (1:500, PK-6105, Lot No. ZK0915, Vector Laboratories). Mouse monoclonal Tau antibody (1:5000, Cat. No. ab246808, Lot No. 1013542-1, Abcam) was used for tau pathology. Sections were incubated with secondary antibodies (1:500, PK-6102, Lot No. ZL0828, Vector Laboratories) and followed by avidin-biotin-peroxidase (1:300) for 2 h at room temperature. The tissue-bound peroxidase was visualized by diaminobenzidine and the nickel reaction (SK-4100, Vector Laboratories). After staining, the sections were dehydrated with alcohol, cleared with xylene and cover slipped on a resinous mounting medium. The density of TH+ and Iba1+ cells was calculated as the number of cells per unit area (1 mm^2^). The “nucleator” probe in the stereology program was used to estimate the mean cross-sectional area of the cells.

### Animal models of narcolepsy: Immunostaining for control and mice models of narcolepsy

We conducted parallel investigations using orexin knockout (OX-KO) mice, orexin-DTA mice, and dog models of narcolepsy (Supplementary Table 3). Animals were housed at a constant temperature of 23 ± 2 °C with lights on at 07.00 am and lights off at 07.00 pm. The orexin-DTA mice were C57BL/6 mice with postnatal ablation of Hcrt neurons, which was induced by doxycycline withdrawal. The experimental group had doxycycline (DOX) food removed for 30 days, followed by a reintroduction of DOX food and euthanasia 14 days later. Control animals had DOX food throughout and were sacrificed at the same time as the experimental group. Control and narcolepsy mouse models were perfused, post-fixed, and transferred through 20% sucrose and 30% sucrose solutions. No antigen retrieval was necessary for the mouse sections. Brainstem sections were cut at 40 µm, and a one-in-three series was analyzed. The sections were blocked in 1% normal goat serum in 0.01 M PBS for 2 h and then incubated with tyrosine hydroxylase (1:10,000, rabbit polyclonal, Cat. No. AB 152, Lot No. 4224636, Millipore Sigma, MO) for 72 h at 4 °C. Sections were subsequently incubated with a secondary antibody (1:500, biotinylated goat anti-rabbit IgG, PK-6101, Lot No. ZL0828 Vector Laboratories) followed by avidin-biotin-peroxidase (1:300, PK-6101, Vector Laboratories) for 2 h each at room temperature. Peroxidase activity was visualized with a diaminobenzidine reaction (SK-4100, Vector Laboratories). Tyrosine hydroxylase-positive (TH+) neurons were bilaterally counted in a one-in-three series, and the count was reported without multiplication by 3. Stereological analysis using the Nucleator probe was performed to assess cell number, distribution, and size, using a Nikon E600 microscope equipped with a three-axis motorized stage, video camera, Neurolucida interface, and Stereo Investigator software (MBF Bioscience, VT).

### Immunostaining for control and narcoleptic dogs

Narcoleptic (Hcrt-R2 mutant) Doberman pinschers and breed-matched controls were studied (Supplementary Table 3). The animals were perfused with saline and formalin, and their brains were removed and stored in formalin prior to sectioning and staining. The formalin-fixed brainstem was infiltrated with 20% and 30% sucrose. Coronal sections (40 µm) were cut on a freezing microtome with one-in-twelve section intervals. One series of sections was stained with cresyl violet (PK501, FD Neurotechnology Inc., Baltimore, MD). The sodium citrate heat antigen retrieval method was used before doing TH immunostaining. For TH immunostaining, data acquisition and analysis followed the same protocol used for mice. TH+ cells were counted bilaterally, and the data was reported as the number of neurons per section.

### Effect of sodium oxybate on norepinephrine and microglia cells in mice

The animals used in this study were related to the research published by Wu et al.^48^. Mice were treated with either saline or sodium oxybate (sodium oxybate oral solution, 500 mg/mL, Xyrem, Jazz Pharmaceuticals) at doses of 150, 300, 600, or 1200 mg/kg, administered intraperitoneally for 14 days (Supplementary Data Table 3). Injections at each dose were given twice daily, at ZT 2 (09:00 am) and ZT 6 (01:00 pm). Two hours after the final dose on the 14th day of treatment, the animals were euthanized with Fatal Plus (100 mg/kg), followed by transcardial perfusion with saline and then 4% paraformaldehyde. The brains were removed from the skull and immersed in 4% paraformaldehyde for 24 h. After fixation, the brains were transferred to 20% sucrose until they sank, then placed in 30% sucrose for 3 days. The brainstems were sectioned into 40 μm slices using a freezing microtome and collected at one-in-three section intervals. For immunohistochemistry, brain sections were washed in PBS and treated with 0.5% hydrogen peroxide for 30 min, followed by PBS washes. Sections were then incubated in blocking solution (1% normal goat serum and 0.3% TritonX-100 in PBS) for 2 h and subsequently incubated with dopamine beta-hydroxylase antibody (1:5000, rabbit monoclonal, Abcam, Cat. No. ab209487) for 72 h at 4 °C. Afterward, sections were incubated with biotinylated goat anti-rabbit IgG (1:500, PK-6101, Vector Laboratories) for 2 h at room temperature, followed by avidin-biotin-peroxidase (1:300, PK-6101, Vector Laboratories) for 2 h. The tissue-bound peroxidase activity was visualized using a diaminobenzidine reaction (SK-4100, Vector Laboratories).

Data analyses were performed by investigators blind to conditions. All staining, counting, and cell measurements were done on coded tissue. In every case, the same individual counted experimental and control tissues in randomized order. Control sections from each brain were processed without the primary antibody and did not show staining. Human brainstem regions and nuclei were identified using the “Atlas of the Human Brain”^82^. For mice, we used “The Mouse Brain in Stereotaxic Coordinates”^83^ and for dogs, the “Stereotaxic Atlas of The Dog’s Brain”^84^. Digital images were captured using a MicroFire camera (Optronics, Goleta, CA) and imported into Corel Draw for contrast and brightness adjustments as needed.

### Statistical analysis

All statistical analyses were performed by SYSTAT Version 13. Data are presented as mean ± S.E.M. The number of subjects in each human and animal study is listed in Table 1, as well as in Supplementary Tables 1–3. A one-way ANOVA was used to assess the dose-response of SXB, followed by Tukey post-hoc comparisons among different doses. Pearson’s correlation coefficient (*r*) was used to examine the relationship between cell loss, disease onset, and duration. Two-group comparisons were made using the *t*-test. All tests were conducted using two-sided analysis at a significance level of 0.05. Sex differences in neuropathology of TH, NM, Hcrt and microglia were statistically analyzed in narcolepsy with cataplexy patients but not in controls due to small sample size.

### Reporting summary

Further information on research design is available in the Nature Portfolio Reporting Summary.

## Supplementary information

Supplementary Information

Description of Additional Supplementary Files

Supplementary Movie 1

## Data Availability

All data are provided in the Source Data file groups https://figshare.com/account/items/31214965. Data is available upon request to the corresponding author after publication.
