# Supplementary material for: Human narcolepsy is linked to degeneration of both locus coeruleus and hypocretin neurons
Source: Nat Commun. 2026 Mar 28;17:4978. doi: 10.1038/s41467-026-70899-x (PMC13237025; doi:10.1038/s41467-026-70899-x)
Supplement: Supplementary file 1 — Supplementary Information [file 41467_2026_70899_MOESM1_ESM.pdf]

Supplementary information

Human narcolepsy is linked to degeneration of both locus coeruleus and hypocretin neurons

NCOMMS-25-28336B- Revision

Supplementary Table 1. Number and size of TH and Iba1 cells in human narcolepsy and control subjects.

Supplementary Table 2. Number and size of hypocretin and microglial cells in the hypothalamus of control and NT1.

Supplementary Table 3. Details of mice and dog data and details of SXB experiment.

Supplementary Figure 1. Hypocretin (orexin) cell loss in DTA mice with 30 days removal of DOX food.

Supplementary Figure 2. Locus coeruleus neurons cease firing during cataplexy in narcoleptic dogs.

Table S1: number and size of TH and Iba1 cells in the LC human narcolepsy and control subjects								
Narcolepsy with cataplexy								
**NT1**	**Age**	**Sex**	**Ctaplexy onset**	**Disease duration (years)**	**TH cell number (1mm²)**	**TH cell size (um²)**	**Iba1 number (1mm²)**	**Iba1 size (um²)**
1	79	M	18	61	92	499.14	56	28.87
2	90	M	18	72	136	548.04	47	34.63
3	79	M	21	58	138	559.03	77	42.16
4	72	M	20	52	128	614.44	50	35.90
5	90	F	18	72	158	547.02	32	30.28
6	94	F	21	73	141	622.54	56	30.63
7	61	F	19	42	163	452.86	55	33.45
8	85	F	23	45	128	585.94	49	28.10
9	88	F	13	70	84	503.61	87	28.16
10	91	F	16	75	122	609.44	53	33.11
11	76	F	30	46	102	604.18	42	32.69
**Control**								
1	71	M			236	525.95	34	26.32
2	82	M			228	440.10	22	28.30
3	80	M			204	485.12	20	27.82
4	83	F			208	461.50	26	23.91
5	72	F			216	499.78	18	15.99
Nt1 - Narcolepsy with cataplexy, Iba1 - ionized calcium-binding adapter molecule, TH - tyrosine hydroxylase								

Table S2:number and size of hypocretin and microglial cells in the hypothalamus of control and NT1

NT1	Age	Sex	Ctaplexy onset	Disease duration (years)	Hcrt cell number/section	Hcrt cell size (um²)	Hcrt % loss	Iba1 number (1mm²)	Iba1 % increase	Iba1 size (um²)
**Narcolepsy with cataplexy**										
1	79	M	18	61	75	259.75	87.02	180	50.00	27.80
2	90	M	18	72	52	242.35	91.03	252	110.00	32.85
3	79	M	21	58	51	348.03	91.18	188	56.66	42.70
4	72	M	20	52	40	229.48	93.07	232	93.33	48.35
5	90	F	18	72	60	247.01	89.61	182	51.66	34.74
6	94	F	21	73	96	296.53	83.38	256	113.33	37.02
7	61	F	19	42	53	303.27	90.92	278	131.67	38.33
8	85	F	23	45	45	227.33	92.21	262	118.34	41.23
9	88	F	13	70	53	200.51	90.82	184	53.33	52.08
10	91	F	16	75	46	190.31	92.03	268	123.40	35.21
11	76	F	30	46	32	321.10	94.46	232	93.32	31.51
**Control**										
1	71	M			502.00	394.66		102		20.70
2	82	M			558.00	334.91		128		28.27
3	80	M			583.00	323.51		134		24.27
4	83	F			634.00	387.09		130		25.73
5	72	F			612.00	323.25		106		19.18

NT1 - Narcolepsy with cataplexy, Hcrt- hypocretin, Iba1 - ionized calcium-binding adapter molecule

Table S3 : TH data from mice and dogs and the effect of sodium oxybate on norepinephrine neurons

TTH+ cells in the LC of mice and dogs

Control	Sex	Age months	TH+ cell number	TH+ cell size (μm^2)
**Mice locus coeruleus TH+ cells**				
1	M	11	916	176.12
2	M	11	851	155.96
3	F	11	868	138.78
4	F	11	906	226.46
5	F	11	933	203.66
**DTA mice Dox food off for 30 days restarted and sacrificed after 14 days**				
1	M	11	861	193.3
2	M	11	893	209.3
3	F	11	882	153.62
4	F	11	869	179.66
5	F	11	813	189.71
**OX-KO mice**				
1	M	6	898	198.46
2	M	6	893	225.91
3	M	6	952	184.34

Dog locus coeruleus TH immunostaining

Control	Sex	Age months	TH+ cell No/section	TH+ cell size (μm^2)
1	M	7	526	428.71
2	M	3	514	402.52
3	M	5	594	427.62
4	F	12	648	405.27
5	F	72	504	412.38
**Narcoleptic dog**				
1	M	32	597	444.09
2	M	33	628	391.64
3	F	6	485	473.42
4	F	5	554	418.91
5	F	15	478	465.3

Effect of sodium oxybate in miceLC DBH+ and Iba1+ cells

C57BL/6J mice for Sodium oxybate study

Saline	Sex	Age monts	DBH+ cell Number (1 mm²)	DBH+ cell size (μm^2)	Iba1+ cell number (1 mm²)	Iba1 cell size (μm^2)
1	M	3	239	170.75	184	44.26
2	M	3	270	157.41	200	39.56
3	M	3	280	191.01	192	45.65
4	M	3	265	197.74	204	38.14
5	M	3	268	184.94	168	29.90
6	M	3	226	182.85	204	33.61
**Sodium oxybate 150mg/kg**						
1	M	3	244	160.06	216	51.16
2	M	3	255	159.85	240	43.68
3	M	3	260	170.51	220	54.54
4	M	3	272	196.14	200	35.42
5	M	3	259	149.78	208	35.70
6	M	3	251	143.89	192	40.43
**300 mg/kg**						
1	M	3	214	141.75	196	37.52
2	M	3	221	169.73	248	38.26
3	M	3	229	166.49	240	55.92
4	M	3	222	138.92	232	42.83
5	M	3	220	151.06	216	46.14
6	M	3	297	168.04	208	43.61
**600 mg/kg**						
1	M	3	228	153.88	240	43.84
2	M	3	245	167.22	192	40.16
3	M	3	258	136.22	248	50.58
5	M	3	241	131.31	256	51.39
6	M	3	211	144.11	236	46.23
**1200 mg/kg**						
1	M	3	177	128.74	244	56.15
2	M	3	118	134.65	252	58.30
3	M	3	215	141.22	288	66.60
4	M	3	232	142.38	224	51.09
5	M	3	296	148.68	256	55.00
6	M	3	278	154.96	244	55.68

DBH - dopamine beta hydroxylase, Iba1 -ionised calcium binding adaptor molecule, OX-KO- orexin knockout mice, SXB - sodium oxybate,TH - tyrosine hydroxylase

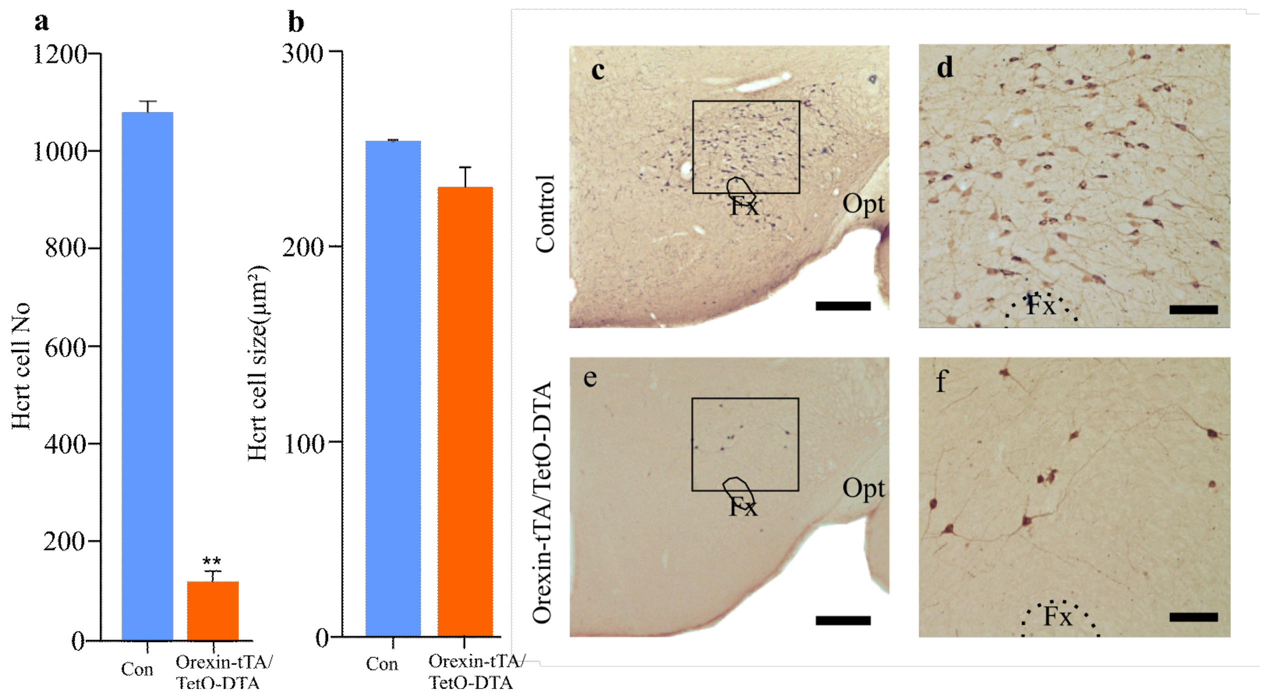

Fig. S1. Hcrt cell loss in DTA mice with 30 days removal of DOX food. **a**,90% Hcrt cell loss in DTA mice (control N = 2, experimental N = 5, t = 25.5, df = 5, P = 0.01). **b**, There is no difference in cell size (t = 1.4, df = 5, P = 0.22). **c** & **d**, Histological images from control. **e** & **f**, Images from DTA mice with hypocretin loss. Scale bar 500 μm (**c** & **e**) and 100 μm (**d** & **f**).

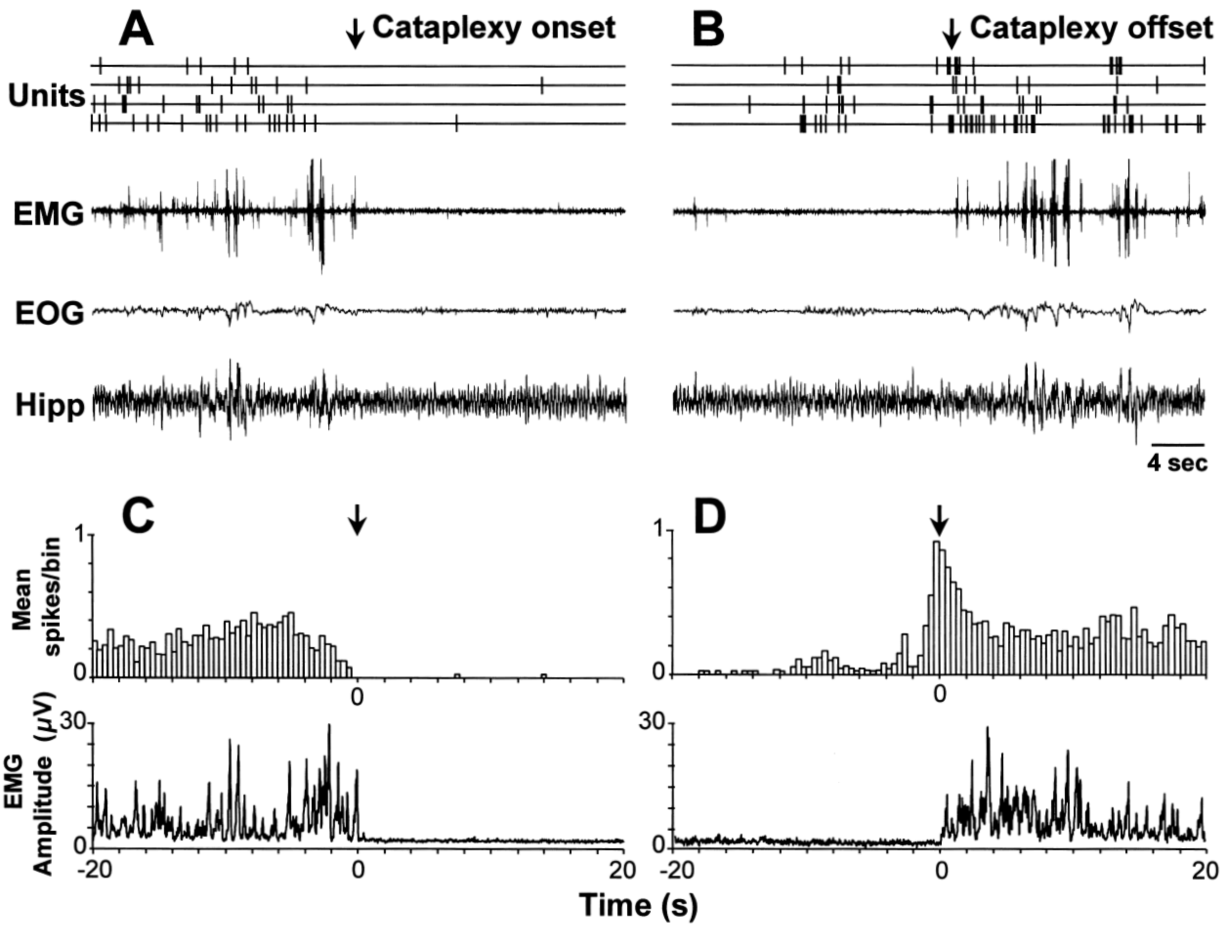

Fig. S2. Locus coeruleus neurons cease firing during cataplexy in narcoleptic dogs (Wu et al., 1999). We previously reported locus coeruleus neurons cease discharge in canine narcolepsy. This finding is consistent with the current anatomical findings.
