# Supplementary material for: Human narcolepsy is linked to degeneration of both locus coeruleus and hypocretin neurons
Source: Nat Commun. 2026 Mar 28;17:4978. doi: 10.1038/s41467-026-70899-x (PMC13237025; doi:10.1038/s41467-026-70899-x)
Supplement: Supplementary file 2 — Description of Additional Supplementary Files [file 41467_2026_70899_MOESM2_ESM.pdf]

Description of Additional Supplementary file

File name: Supplementary Movie 1

Activity of a hypocretin neuron in sleep and waking states. Activity is maximal during presumed “pleasurable” activities including forward movement, grooming and “relaxed” eating, but is minimal when the rat is startled by awakening to food presentation, despite arousal and activated electroencephalogram. (Ref. 51).
